# Extract from Clinical Lecture, Primary Dentition

**Published:** 1890-01

**Authors:** Louis Starr


### Extract from Clinical Lecture—Primary Dentition.

BY DR. LOUIS STARR, M.D.

Clinical Professor of Diseases of Children in the Hospital of the University of Pennsylvania, etc.

The advance of the two incisor teeth in the case I have shown you, have hinted to me that it would be well to occupy the remainder of this hour with a few remarks upon the subject of primary dentition.

You know that normally the human being cuts two sets of teeth, the primary or milk teeth—so-called from their whiteness—and the permanent teeth.

Primary dentition is usually performed between the fourth and thirtieth months. The teeth in this set are twenty in number, and are cut in groups, a period of rest occurring between the eruption of each group. The first teeth to appear are the two central incisors of the lower jaw, usually at the sixth or seventh month, though at times as early as the fourth. Then there is an interval of from three to nine weeks, followed by the eruption of the second group, the four incisors of the upper jaw, between the eighth and tenth months. This is followed by an interval of from six to twelve weeks, when the third group appears, the two lateral incisors of the lower jaw and the four first molars, between the twelfth and fifteenth months. After a rest of twelve weeks, the fourth group appears, the canine or eye and stomach teeth, between the eighteenth and twenty-fourth month, and this is followed by a period of from four to twelve weeks before the appearance of the fifth group, the four last teeth or posterior molars, by the thirtieth month.

The period of rest is a very necessary and important one. During dentition there is a disturbance of the whole system, and should all the teeth be cut at once the child would be made very ill. Therefore this period is required for recuperation.

The normal plan of eruption may be deviated from. The teeth may be cut too early. I have seen cases in which the first group of teeth was present at birth, while it is not unusual to see them by the fourth or fifth month. Nor is this an unfavorable circumstance, for an early dentition is usually an easy one. It is more apt to occur in childreu fed from the breast, and in girls than in boys. Next, dentition may be delayed, and the first teeth not appear until the eighth or ninth month, nor the last group before the third year. This delay in dentition should always bring up the question of rickets. It is more apt to occur in bottle-fed babies. Again, the teeth may be cut irregularly, the groups not appearing in their normal sequence, or a number may appear at the same time. Delayed and irregular dentitions are both apt to be hard, and to give rise to certain symptoms of disease. The teeth most apt to give trouble are the third and fourth groups, but it is a fact that if one group causes a great deal of trouble, the remainder are apt to be cut without difficulty. The symptoms attending dentition are as follows: When you examine the jaws of a child in whom the teeth are not advancing you will observe that the gums are of pale pink color and have a well-marked ridge-like margin. When the teeth advance this ridge disappears, the gums become swollen, reddened, and moderately hot and tender to the touch. There is some increased salivation and the gum is the seat of moderate pain. These features of the normal process continue until the tooth pierces the mucous membrane, but are never so severe as to cause any disturbance to the child.

In difficult dentition all of these normal symptoms are exaggerated, so that we may have catarrhal or aphthous stomatitis, with pain, fever, loss of appetite, and general malaise. Other local conditions are ulceration on either side of the frænum linguæ from friction, from the sharp edges of the lower incisors and profuse salivation. The latter condition is an important one for it may lead to so much wetting of the garments as to produce and maintain a severe bronchial catarrh, which, by the way, is apt to resist medical treatment unless the clothing is kept dry by a bib of oil silk or rubber cloth.

The general conditions attending difficult dentition are enlargement of the lymphatic glands at the angle of the jaw, certain eruptions upon the skin, especially eczema, strophulus, and urticaria; vomiting, diarrhœa, infantile convulsions, so-called dental paralysis—the latter being probably a condition of anterior polio-myelitis; blenorrhœa and otorrhœa are also common complications.

Many authors have denied the connection of these symptoms with the eruption of the teeth, as they regard dentition as a physiological process, and therefore incapable of inducing symptoms of disease. I think, however, there is an undoubted connection, for the interval between the fourth and thirtieth months of an infant's life—the period of primary dentition—is a year of great and widely distributed progress. Not only are the teeth advancing but the follicular apparatus of the stomach and intestinal canal is undergoing development in preparation for the digestion and absorption of mixed food, the cerebro-spinal system is rapidly growing and functionally very active, and the organs and tissues of the whole body are in a state of rapid change. This period of normal transition must also be one in which there is great susceptibility to disease, provided there be a causal influence at work. Such an influence may either originate outside of the body or come from within in the form of some physiological process. Difficult dentition, in my mind, stands prominent in the latter class.

Whichever of the above symptoms may be present, appropriate medical treatment is of course indicated, but I would especially call your attention to the propriety of lancing the gums over advancing teeth so as to thoroughly free them and do away with backward pressure and consequent irritation of the delicate nervous system. One should not wait until the gums are soft and swollen and until the edge of the tooth can be seen through the mucous membrane, but should cut freely down, so as to divide the denser layers of the gum and connective tissues whenever any of the complications I have mentioned are present, and examination of the gums shows advancing teeth. The operation is never properly performed unless the edge of the lancet can be felt to grate against the tooth. The incision over incisor teeth should be linear, over the canines rectangularly crucial, and over the molars obliquely crucial.—*Med. and Surgical Reporter.*

